# Locally adapted fish populations maintain small-scale genetic differentiation despite perturbation by a catastrophic flood event

**DOI:** 10.1186/1471-2148-10-256

**Published:** 2010-08-23

**Authors:** Martin Plath, Bernd Hermann, Christiane Schröder, Rüdiger Riesch, Michael Tobler, Francisco J García de León, Ingo Schlupp, Ralph Tiedemann

**Affiliations:** 1 Department of Ecology & Evolution, J.W. Goethe University Frankfurt, Siesmayerstrasse 70-72, D-60054 Frankfurt am Main, Germany; 2 Institute of Biochemistry & Biology, Unit of Evolutionary Biology/Systematic Zoology, University of Potsdam, Karl-Liebknecht Str. 24-25, D-14476 Potsdam, Germany; 3 Department of Zoology, University of Oklahoma, Norman, OK 73019, USA; 4 Department of Biology and Department of Wildlife and Fisheries Sciences, Texas A&M University, 2258 TAMU, College Station, TX 77843, USA; 5 Laboratorio Genética para la Conservación, Centro de Investigaciones Biológicas del Noroeste, S. C., Mar Bermejo No. 195, Col. Playa Palo de Santa Rita, A. P. 128, La Paz, Baja California Sur 23090, Mexico

## Abstract

**Background:**

Local adaptation to divergent environmental conditions can promote population genetic differentiation even in the absence of geographic barriers and hence, lead to speciation. Perturbations by catastrophic events, however, can distort such parapatric ecological speciation processes. Here, we asked whether an exceptionally strong flood led to homogenization of gene pools among locally adapted populations of the Atlantic molly (*Poecilia mexicana*, Poeciliidae) in the Cueva del Azufre system in southern Mexico, where two strong environmental selection factors (darkness within caves and/or presence of toxic H_2_S in sulfidic springs) drive the diversification of *P. mexicana*. Nine nuclear microsatellites as well as heritable female life history traits (both as a proxy for quantitative genetics and for trait divergence) were used as markers to compare genetic differentiation, genetic diversity, and especially population mixing (immigration and emigration) before and after the flood.

**Results:**

Habitat type (i.e., non-sulfidic surface, sulfidic surface, or sulfidic cave), but not geographic distance was the major predictor of genetic differentiation. Before and after the flood, each habitat type harbored a genetically distinct population. Only a weak signal of individual dislocation among ecologically divergent habitat types was uncovered (with the exception of slightly increased dislocation from the Cueva del Azufre into the sulfidic creek, El Azufre). By contrast, several lines of evidence are indicative of increased flood-induced dislocation within the same habitat type, e.g., between different cave chambers of the Cueva del Azufre.

**Conclusions:**

The virtual absence of individual dislocation among ecologically different habitat types indicates strong natural selection against migrants. Thus, our current study exemplifies that ecological speciation in this and other systems, in which extreme environmental factors drive speciation, may be little affected by temporary perturbations, as adaptations to physico-chemical stressors may directly affect the survival probability in divergent habitat types.

## Background

### Ecological speciation

Ecological speciation describes the process during which reproductive isolation evolves as a result of ecologically based divergent selection [1-3]. Evidence for ecological speciation comes from theoretical considerations, empirical studies in natural systems, and from laboratory evolution experiments [3-6]. Most studies on ecological speciation in animals focused on biotic selective agents causing divergence among populations, for example in populations with differences in resource use [7-10], differences in predation risk [11,12], or exposure to different kinds of parasites [13-15]. While the literature on ecological speciation in response to abiotic selective agents and stressors is extensive for plants [16-20], only few studies to date investigated ecological speciation along abiotic environmental gradients in animals [21,22]. Although stressful environments have long been known to be associated with bouts of directional selection [23], the concept of stress and the maintenance of homeostasis through adaptation is often ignored in the study of speciation in animals and more emphasis is put on its role in population decline and extinction [24]. Here we investigate a system where adaptation to ecologically divergent habitat patches--with presence or absence of abiotic stressors--drives the divergence among locally adapted populations of a small Mexican freshwater fish, the Atlantic molly (*Poecilia mexicana*, Poeciliidae) [25-29].

### Poecilia mexicana in the Cueva del Azufre System

**Figure 1:**
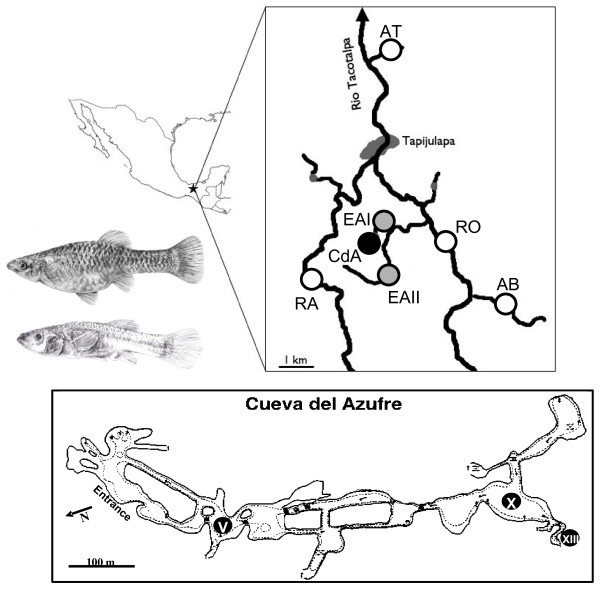
**Overview sketch of the Cueva del Azufre system**. *Poecilia mexicana *populations sampled for the present study originated from non-sulfidic surface sites (white circles), Arroyo Bonita (AB), Arroyo Tres (AT), Río Oxolotan (RO) and Río Amatan (RA), sulfidic surface sites (gray circles), El Azufre I and II (EA I, EA II) and the sulfidic Cueva del Azufre (CdA; black circle). In Tapijulapa, Río Amatan joins Río Oxolotan to form the Río Tacotalpa. Below: overview of the Cueva del Azufre with different cave chambers sampled for this study (V, X, and XIII). Insert: surface (above) and cave dwelling *P. mexicana *female.

In the Cueva del Azufre system in southern Mexico, ecologically divergent aquatic habitats are characterized by the presence or absence of toxic hydrogen sulfide (H_2_S) and/or light (*i.e*., cave versus surface habitats [26,27,30]), resulting in several non-toxic surface habitats, a toxic creek (El Azufre), as well as a toxic and a non-toxic cave: the 'Cueva del Azufre' (also called 'Cueva de Villa Luz' or 'Cueva de las Sardinas' [31]), and the 'Cueva Luna Azufre', respectively ([26,32]; Fig. 1). The hydrogen sulfide in this system stems from natural (volcanic) sources [33]. Both the presence of H_2_S and the absence of light exert strong selection on organisms exposed to them. Hydrogen sulfide is acutely toxic to most metazoans and leads to extreme hypoxia in the water [34-36]. The absence of light in caves inhibits the use of visual senses, and cave-dwellers are under selection to cope with the impossibility for visual orientation and communication [37-40], especially if they evolved from a diurnal surface-dwelling form like in *P. mexicana *(Poeciliidae) [41,42] and *Astyanax mexicanus *(Characidae, [43-45]).

No major physical barriers prevent fishes in the Cueva del Azufre system from moving between different habitat types that are only a few hundred meters apart (Fig. 1); still, only *Poecilia mexicana *has successfully colonized all of these different habitats [26,30,31,46,47]. Although locally adapted populations can be crossbred in the laboratory [48], remarkably strong genetic differentiation has been uncovered at molecular markers (microsatellites and cytochrome *b*: [25,26]), and habitat type is the strongest predictor of genetic similarity among populations. Several studies reported on site-specific adaptive trait divergence in morphological [26,49,50], physiological [28,29], behavioral [42,46,48,51,52], and life history traits [53-56]. Altogether, this suggests that adaptation to divergent environmental conditions drives genetic differentiation in this system. Natural selection against immigrants from ecologically divergent populations plays a major role in stabilizing the observed small-scale genetic structuring ([27,57]; see discussion for details), and female mate discrimination against divergent male phenotypes also plays a role [27].

### The 2007 flood as a natural experiment

**Figure 2:**
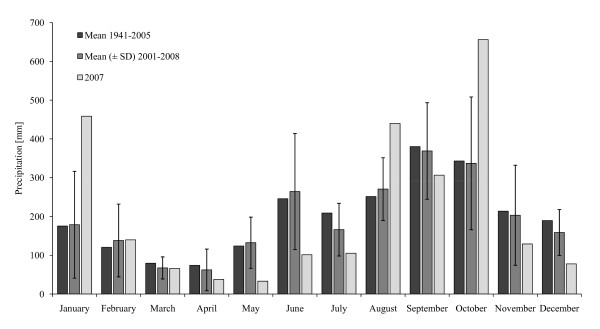
**Mean monthly precipitation for the state of Tabasco from 1941-2005, 2001-2008, and for 2007 based on data obtained from Servicio Meterológico Nacional (México) **[59]. Measures of variance could only be obtained for the period of 2001-2008, but note that mean monthly rainfalls for this period closely match long-term means (from 1941-2005). The 2007 floods occurred in late October and early November, which coincided with a peak in rainfall in October.

At the end of October and beginning of November of 2007, the rivers around the Cueva del Azufre system and the state of Tabasco in general were subject to intense flooding, which resulted from heavy rainfalls generated by two subsequent cold fronts moving into the area within a nine day period (with locally > 1500 mm of precipitation during the first rainfall episode and > 1100 mm during the second one; [58]). Taken together, these have been considered as the most intense rainfalls in more than 50 years. Overall, rainfall in Tabasco was 82% above the long-term average of October and five times as high as the historical average over a period of 24 hours ([59]; see also Fig. 2 for historic and 2007 rainfall patterns). These high rates of rainfall led to the flooding of more than 60% of the state of Tabasco and water discharges of up to 8,000 m^3^/s in the Río Grijalva system, which the Cueva del Azufre system is a part of. According to reports from the local population, water levels in the Ríos Amatan and Oxolotán, the two major rivers around the Cueva del Azufre system, rose up to 8 m above the normal water level.

In aquatic ecosystems, the effects of small-scale floods, which are often common, seasonal events, have been well documented; not only do they reshape the physical environment, but they also influence fish community structure [60-65]. However, the impacts of larger floods are less well understood (*e.g*., [66]). Severe floods are not only associated with peaks in flow conditions within the riverbed, but also with a lateral expansion of the water body. Therefore, while seasonal floods are fundamental for the maintenance of biodiversity in floodplains [67,68], larger floods are thought to have an opposite impact at least on headwater habitats (see [69,70] for discussion). Here, floodwaters can displace individuals and wash them downstream, potentially leading to drastic reductions in local population sizes, genetic bottlenecks, and even local population extinction [69,71], if upstream populations are only subjected to downward drift without counteracting upward colonization movements (*i.e*., Müller's "drift paradox" [72]).

In this study, we were particularly interested in how the catastrophic flood affected genetic structure and life history trait divergence in parapatric, locally adapted *P. mexicana *populations. The flooding of the water bodies around the Cueva del Azufre system presumably led to temporarily increased connectivity among habitat patches (as commonly observed by the authors after heavy rainfalls during the rainy season). Two effects are likely to have occurred due to the flood: (*i*) Increased flow regimes could have led to displacement of locally adapted *P. mexicana *to habitat patches further downstream (*e.g*., washing cave fish from the cave to the outside or fish from the sulfidic into the non-sulfidic habitats), and (*ii*) the flood waters were likely to have temporarily diluted the hydrogen sulfide concentrations, potentially enabling immigration by fish from the non-sulfidic habitats. Hence, small-scale genetic structure caused by local adaptation to H_2_S levels could have been lost, or at least reduced due to admixture of gene pools. As a result, this could lead to a reversal of the observed processes of differentiation due to hybridization/homogenization, as has been observed between different ecotypes in other systems [6,73,74]. Alternatively, natural selection in this system might be strong enough to maintain population-specific differences despite severe temporal disturbances. Hence, to test whether the Tabasco flood led to homogenization of locally adapted populations, we compared patterns of genetic differentiation, genetic diversity, and especially genetically detectible individual dislocation before and after the flood. First, we used a population genetic approach based on allelic variation of nine neutral nuclear markers (microsatellites). Secondly, we used a quantitative genetic approach focusing on life history traits as markers.

## Methods

### Study system and sample origin

**Figure 3:**
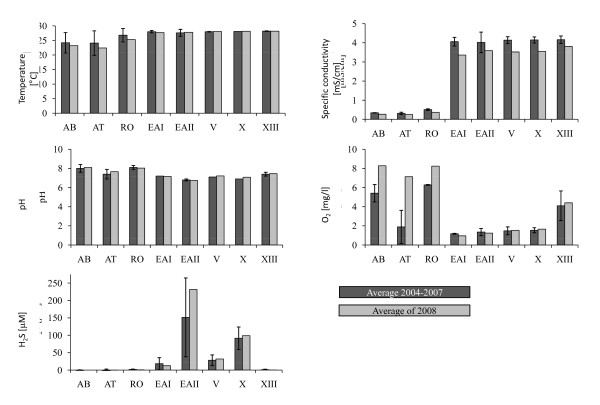
**Measurements of temperatures, specific conductivity, pH, oxygen concentrations, and H_2_S concentrations pre- and post-flood**. Temperature, specific conductivity, pH, and oxygen content were measured using a Hydrolab Multisonde 4A (Hach Environmental). Measurements and calibration of probes were conducted according to the manufacturer's recommendations. For the measurement of H_2_S concentrations, 1 ml of water was injected into a vial containing 1 ml of zinc acetate (0.12 M with 0.5 ml NaOH 1.5 M in a N_2_-atmosphere) using a syringe. The vials were stored at room temperature and photometric measurements were conducted in the laboratory according to [76]. Pre-flood data were previously published in [26] and available from visits in three (AB, AT, EAII) or four (RO, EAI, V, X, XIII) different years. Values of each variable were averaged for each year (at least three Hydrolab readings and one H_2_S sample was taken at each site and visit) and mean and standard deviation among years are shown. Post-flood data were collected in January 2008. Depicted are mean values based on at least two Hydrolab readings and two H_2_S samples taken at each site and visit.

The Atlantic molly, *Poecilia mexicana*, is a widespread freshwater fish occurring along the Atlantic versant of Mexico, where it can be found in various streams, lakes, and coastal lagoons [75,76]. Our study sites are located near the village of Tapijulapa in the southern Mexican state of Tabasco (Fig. 1). We sampled three different habitat types that are characterized by the presence or absence of H_2_S and/or light (see Fig. 3 for a comparison of site-specific selection pressures by abiotic stressors before and after the flood). All sites are connected by watercourses located within 10 km of each other (river distance), and the average distance between sites is about 3.5 km (Table 1). Sites sampled include normal (non-sulfidic, surface) rivers (*N *= 3 sites), a sulfidic surface creek (*N *= 2 sites), and a sulfidic cave (Cueva del Azufre), in which we sampled from multiple (*N *= 3) cave chambers (V, X, and XIII; [31]). The non-sulfidic cave (Cueva Luna Azufre) was not accessible after the flood. Inside the Cueva del Azufre, several springs discharge water rich in hydrogen sulfide (H_2_S), so cave mollies not only live in darkness [77], but also have to cope with the adverse effects of a naturally occurring toxicant [30] and thus face two strong selective forces [25,26]. In addition, the abiotic differences among habitat types are correlated with differences in biotic environmental conditions, such as resource availability [78], predators [47,79-81], and parasites ([82]; M. Tobler et al., unpublished data).

**Table 1:** Distances between sites in km.

	AB	AT	RO	EA1	EA2	V	X	XIII
AT	8.50							
RO	2.52	5.98						
EAI	4.10	6.72	1.58					
EAII	4.60	7.22	2.08	0.50				
V	4.26	6.88	1.74	0.16	0.66			
X	4.36	6.98	1.84	0.26	0.76	0.10		
XI	4.40	7.02	1.88	0.30	0.80	0.14	0.04	
XIII	4.41	7.03	1.89	0.31	0.81	0.15	0.05	0.01

Distances were estimated by plotting GPS coordinates of the collection sites into GoogleEarth and then measuring the river-distance between sites with the measurement tool.

The water from the cave eventually drains into the El Azufre (EA I), which is a sulfidic surface creek also containing high concentrations of hydrogen sulfide. In addition to sulfidic discharge from the cave, there are several independent sulfidic springs upstream of the confluence with the creek leaving the Cueva del Azufre (EA II). The El Azufre meanders for approximately 1.5 km and eventually drains into the Río Oxolotán (RO; [26]; Fig. 1). Besides these divergent habitat types, we sampled non-sulfidic surface habitats, including the Arroyo Bonita (AB) and Arroyo Tres (AT). Eventually, all habitats sampled drain into Río Oxolotán.

Pre-flood samples were available from January 2006 and May/June 2007 [26]; our post-flood samples were collected in January 2008 (2 months after the flood). Because habitat structures differed between sampling sites, different capture methods were employed. In the cave, where the water is very shallow and low ceilings preclude seining, fish were caught with dip nets (13 × 14 cm, 1 mm mesh-width). In the other habitats, fish were caught using a seine (4 m long, 4 mm mesh-width). Fin clips for extraction of DNA were stored in 96% ethanol at 4°C. Between 18 and 24 fish from each site were genotyped before the flood and 8 to 33 individuals after the flood (Tables 2 and 3). Post-flood sample sizes varied depending on the accessibility of sites and the abundance of fish after the flood. All specimens used for life history trait characterization were euthanized using MS222 immediately after capture and fixed in a 10% formaldehyde solution.

**Table 2:** Genetic diversity in surface-and cave-dwelling Atlantic mollies (*Poecilia mexicana*) before the catastrophic 2007 flood.

Locus	Number of alleles	Range of allele size	Test	AB (*N *= 24)	AT (*N *= 19)	RO (*N *= 24)	EAI (*N *= 20)	EAII (*N *= 20)	V (*N *= 18)	X (*N *= 20)	XIII (*N *= 18)
GA-I 29B	12	217-255 (217-253)	*H*_o_	0.17	0.53	0.46	0.35	0.65	0.00	0.20	0.00
			*H*_E_	0.23	0.51	0.53	0.40	0.51	0.00	0.19	0.00
			*A*	2.89	4.55	4.55	2.00	2.40	1.00	2.29	1.00
GA-IV 42	45	205-463 (227-463)	*H*_o_	0.92	0.95	0.79***	0.65	0.70	0.61	0.70	0.56
			*H*_E_	0.92	0.90	0.90	0.77	0.68	0.76	0.73	0.52
			*A*	10.95	11.02	9.94	5.88	4.72	6.29	6.12	4.08
GT-II 33	10	173-231 (175-205)	*H*_o_	0.63	0.53	0.75	0.05	0.00	0.06	0.00	0.00
			*H*_E_	0.62	0.65	0.69	0.05	0.00	0.05	0.00	0.00
			*A*	3.82	4.21	4.46	1.40	1.00	1.44	1.00	1.00
GA-II 41	11	122-142 (130-142)	*H*_o_	0.67	0.72	0.83	0.55	0.50	0.61	0.05	0.11
			*H*_E_	0.58	0.64	0.64	0.57	0.56	0.44	0.05	0.28
			*A*	4.27	3.87	4.48	3.68	3.05	2.44	1.40	1.98
GA-I 29A	15	214-258 (239-258)	*H*_o_	0.67	0.72	0.83	0.80	0.85	0.78	0.70	0.61
			*H*_E_	0.66	0.68	0.72	0.77	0.75	0.66	0.65	0.53
			*A*	4.79	5.18	5.65	4.75	4.71	3.88	4.48	2.44
GA-V 18	19	113-153 (119-153)	*H*_o_	0.96	0.79	0.88	0.55*	0.35***	0.41	0.35	0.44
			*H*_E_	0.88	0.89	0.84	0.59	0.47	0.50	0.49	0.45
			*A*	8.58	9.35	7.43	3.28	2.65	2.00	2.40	3.99
GA-I 26	33	167-281 (167-281)	*H*_o_	0.96	0.74	0.83	0.65	0.25	0.24	0.80	0.11
			*H*_E_	0.89	0.87	0.92	0.86	0.56	0.27	0.89	0.10
			*A*	10.20	8.84	10.60	8.93	5.23	3.14	9.62	1.70
GA-III 28	31	196-264 (196-262)	*H*_o_	0.86	0.68	0.92	0.90	0.67	0.65***	0.75	0.44
			*H*_E_	0.82	0.78	0.88	0.87	0.86	0.79	0.83	0.50
			*A*	7.93	6.80	8.81	9.04	8.25	7.84	7.94	5.40
GT-I 13B	3	217-237 (217-237)	*H*_o_	0.21	0.00	0.29	0.00	0.00	0.12	0.00	0.00
			*H*_E_	0.26	0.00	0.32	0.00	0.00	0.11	0.00	0.00
			*A*	2.53	1.00	2.70	1.00	1.00	1.00	1.73	1.00
Mean across loci			*H*_o_	0.67	0.63	0.73	0.50	0.44	0.39	0.39	0.25
			*H*_E_	0.65	0.66	0.72	0.54	0.49	0.40	0.43	0.26
			*A*	6.22	6.09	6.51	4.44	3.67	3.23	4.11	2.51

For each population and locus, observed (*H*_o_) and expected (*H*_E_) heterozygosities as well as allelic richness (*A*) are given. Zero values indicate that the locus is monomorphic in this population. Ranges of allele sizes are given for the entire data set (upper values) and within a single individual (the lower value in parentheses gives the largest observed range). Non-sulfidic surface habitats: AB, Arroyo Bonita, AT, Arroyo Tres, RO, RÍo Oxolotan; sulfidic surface sites: EAI, El Azufre I, EAII, El Azufre II; sulfidic cave (Cueva del Azufre), chambers V, × and XIII. Deviation from HWE after Bonferroni correction (i.e., heterozygote deficiency) are indicated by asterisks, whereby *P < 0.05, ** *P *< 0.001, and *** *P *< 0.001.

**Table 3:** Genetic diversity in surface- and cave-dwelling Atlantic mollies (*Poecilia mexicana*) after the 2007 flood.

Locus	Number of alleles	Range of allele size	Test	AB (*N *= 16)	AT (*N *= 14)	RO (*N *= 33)	EAI (*N *= 31)	EAII (*N *= 27)	V (*N *= 25)	X (*N *= 19)	XIII (*N *= 8)
GA-I 29B	13	217-255 (217-255)	*H*_o_	0.44**	0.36***	0.33***	0.32	0.33	0.28	0.26	0.38
			*H*_E_	0.65	0.67	0.39	0.31	0.40	0.28	0.28	0.32
			*A*	6.22	4.12	3.95	1.98	2.00	2.56	2.57	3.00
GA-IV 42	39	187-403 (215-357)	*H*_o_	0.5***	0.71**	0.53***	0.81	0.44	0.52***	0.68	0.38
			*H*_E_	0.90	0.86	0.82	0.76	0.68	0.62	0.70	0.57
			*A*	10.38	10.44	8.54	6.56	4.74	5.88	5.08	5.00
GT-II 33	11	173-229 (175-229)	*H*_o_	0.75	0.43	0.67	0.03	0.00	0.00	0.00	0.13
			*H*_E_	0.72	0.53	0.64	0.03	0.00	0.00	0.00	0.12
			*A*	4.75	3.14	3.85	1.26	1.00	1.00	1.00	2.00
GA-II 41	13	122-146 (130-146)	*H*_o_	0.63	0.71	0.45	0.52	0.70	0.24	0.21	0.25
			*H*_E_	0.60	0.77	0.54	0.56	0.60	0.21	0.19	0.23
			*A*	4.93	5.98	4.19	3.36	3.47	1.92	1.90	3.00
GA-I 29A	15	214-258 (214-244)	*H*_o_	0.50	0.64	0.61	0.84	0.48	0.56	0.53	0.38
			*H*_E_	0.57	0.61	0.66	0.75	0.65	0.55	0.57	0.51
			*A*	3.50	3.65	4.41	4.65	3.99	2.86	3.51	3.00
GA-V 18	20	115-157 (119-155)	*H*_o_	0.81	0.92	0.79	0.48***	0.22***	0.48	0.47***	0.75
			*H*_E_	0.88	0.89	0.82	0.52	0.25	0.49	0.43	0.54
			*A*	8.80	10.05	6.72	2.96	1.95	2.54	3.32	3.00
GA-I 26	37	147-257 (173-237)	*H*_o_	0.80	0.93	0.58	0.67	0.67	0.88	0.63	0.75
			*H*_E_	0.87	0.87	0.83	0.91	0.85	0.92	0.87	0.84
			*A*	9.15	10.06	7.41	9.73	9.09	10.58	8.61	8.00
GA-III 28	30	202-288 (218-288)	*H*_o_	0.81	0.93	0.91	0.90	0.74	0.84	0.89	0.88
			*H*_E_	0.82	0.82	0.81	0.85	0.86	0.81	0.86	0.83
			*A*	8.28	8.01	7.60	8.66	8.50	7.67	7.96	9.00
GT-I 13B	6	215-237 (215-237)	*H*_o_	0.63	0.07	0.00	0.03	0.04	0.00	0.05	0.25
			*H*_E_	0.45	0.07	0.00	0.03	0.04	0.00	0.05	0.22
			*A*	2.50	1.57	1.00	1.26	1.30	1.00	1.42	2.00
Mean across loci			*H*_o_	0.65	0.63	0.54	0.51	0.40	0.42	0.41	0.46
			*H*_E_	0.72	0.68	0.61	0.52	0.48	0.43	0.44	0.46
			*A*	6.50	6.34	5.30	4.49	4.00	4.00	3.93	4.22

For each population and locus, observed (*H*_o_) and expected (*H*_E_) heterozygosities as well as allelic richness (*A*) are given. Zero values indicate that the locus is monomorphic in this population. Ranges of allele sizes are given for the entire data set (upper values) and within a single individual (the lower value in parentheses gives the largest observed range). Non-sulfidic surface habitats: AB, Arroyo Bonita, AT, Arroyo Tres, RO, RÍo Oxolotan; sulfidic surface sites: EAI, El Azufre I, EAII, El Azufre II; sulfidic cave (Cueva del Azufre), chambers V, × and XIII. Deviation from HWE after Bonferroni correction (i.e., heterozygote deficiency) are indicated by asterisks, whereby *P < 0.05, ** *P *< 0.001, and *** *P *< 0.001.

The research followed internationally recognized guidelines and applicable national legislation. We received ethical approval from the deputy of animal welfare of the University of Potsdam.

### Microsatellite analysis

DNA was extracted from tissue samples using the DNeasy DNA Extraction kit (QIAGEN, Hilden, Germany) according to the manufacturer's recommendations. Between 36 and 576 ng (mean ± S.E.: 187 ± 115 ng) of genomic DNA were used as template for each PCR. Ten microsatellite loci were amplified in five (partly multiplex) PCR according to the cycling parameters described in [83] but with modified annealing temperatures to accommodate for multiplexing: (1) GAIV42, GAI29B and GTII33: 3 cycles at 52.3°C and 37 cycles at 49.3°C, (2) GAI29A and GAII41: 3 cycles at 56.4°C and 37 cycles at 53.4°C, (3) GAI26 and GAIII28: 3 cycles at 55.6°C and 37 cycles at 52.6°C, (4) GAV18 and GTI49: 3 cycles at 48.5°C and 37 cycles at 45.5°C. One locus (GTI13b) was amplified separately, because multiplex PCR did not yield satisfying results. For this marker, two-step amplification was performed with 3 cycles at 55.6°C, followed by 37 cycles at 52.6°C. We initially included the locus GTI49 [25], but the results were ambiguous for a considerable number of individuals (*i.e*., in some cases more than two peaks were detected). Therefore, it was excluded from the analysis. Fragment sizes were determined on an ABI 3700 automatic sequencer using GENEMAPPER 3.7 and an internal size standard (500 LIZ, Applied Biosystems).

Data for *N *= 163 individuals (collected before the flood) were re-analyzed from a previous study [25], and *N *= 173 additional specimens (after the flood) were genotyped for this study. We tested for the independent inheritance of all loci (linkage disequilibrium) with a likelihood ratio test using GENEPOP on the web [84]; as reported before [25], no indication of linkage was detected for the loci employed. FSTAT [85] was used to calculate observed (*H*_O_) and expected heterozygosity (*H*_E_), to conduct a probability test for deviations from Hardy-Weinberg equilibrium (HWE), and to calculate allelic richness (*A*). To compare the genetic variability of the examined populations before and after the flood, locus-wise values for *H*_O_, *H*_E_, and *A *were subjected to paired *t*-tests for each population separately.

We also used FSTAT to calculate pair-wise genetic distances (*F*_ST_), and *P*-values were based on 1,000 permutations. The proportion of the intra- and inter-population variance was calculated with an AMOVA [86] as implemented in ARLEQUIN [87]. We tested whether genetic differentiation after the flood was correlated with the differentiation prior to the flood by comparing pair-wise genetic distances using a Mantel test with 2,000 randomization as implemented in FSTAT [85]. Moreover, we tested for the effects of habitat type (same or different; *i.e*., 'isolation-by-adaptation'; [5,88]) and geographic distance between sites (*i.e*., 'isolation-by-distance'; [89]) on genetic differentiation pre- and post-flood by separately subjecting pre and post pair-wise *F*_ST_-values to partial Mantel tests.

To calculate first-generation migrants within each data set (before and after the flood), we employed GENECLASS 2.0 [90] using the L_home likelihood computation, the Bayesian method of classification [91], and a threshold *P*-value of 0.05. We calculated the proportion of inferred migrants as a fraction of all specimens sampled in the respective population. If we assume that migrants and residents have the same probability to be sampled, this proportion should provide an unbiased estimator for the percentage of migrants in the population. As for pair-wise genetic distances, we used a Mantel test to correlate relative numbers of post-flood migrants (as a fraction of migrants in the 'recipient' population) with relative numbers of pre-flood migrants (arcsine square root-transformed). Also, we used a partial Mantel test to compare the relative number of migrants between pair-wise sites prior and after the flood (see [26,70]). Predictor matrices were based on distance between sites and habitat type (same or different). To test whether abiotic environmental conditions affected the directionality of first generation migrants, we also included matrices describing the difference in habitat types with respect to the presence of H_2_S (-1: movement from a sulfidic to a non-sulfidic habitat; 0: no change; +1: from non-sulfidic to sulfidic) and the absence of light (-1: movement from a cave to a surface habitat; 0: no change; +1: from surface to cave).

STRUCTURE version 2.3.2 Beta [92,93] was employed to identify the number of genetically distinct clusters (*k*) according to HWE and linkage equilibrium for each data set (before and after the flood) with the method presented by Evanno et al. [94]. For each value of *k*, three iterations were run using the admixture model, with a burn-in period of 10,000, followed by 90,000 iterations for values of *k *= 1 through 8. Each simulation was performed using an ancestry model incorporating admixture, a model of correlated allele frequencies, and the prior population information.

### Life history trait characterization

Female reproductive life history traits show pronounced differences across habitat types [54,56]; in particular differences across populations in offspring size and fecundity were found to have a strong genetic basis in this system [53,55]. Most importantly, female life history traits are site-specific (while male life history traits are slightly more variable: R. Riesch, unpublished data) and thus allow correctly assigning individuals to their population of origin [56]. Therefore, we utilized female life history traits as another means of testing for potential dislocation of individuals by the flood.

Life history data were reanalyzed from a previous publication [56]. Pre-flood collections were made in May 2007 from (*a*) the Cueva del Azufre (chambers V and X), (*b*) El Azufre (sites I and II), and (*c*) the Río Amatan (RA), a river that joins the Río Oxolotán approximately 3 km downstream of the El Azufre/Río Oxolotán confluence ([26]; Fig. 1). Due to the flooding, this particular site was inaccessible in January 2008. Hence, our 2008 (post-flood) collections stem from (*a*) the Cueva del Azufre (chambers V and X), (*b*) El Azufre (I and II), and (*c*) Arroyo Bonita (AB). Following standard life history protocols (see [56] for details), we assessed the following life history traits: standard length [mm], female lean weight [g], female fat content [%, a measure for female condition], fecundity [number of offspring], embryo dry weight [mg], embryo fat content [%, a measure for offspring condition], and embryo developmental stage. Furthermore, to evaluate female investment in reproduction, we calculated reproductive allocation [%] by dividing offspring weight by the sum of offspring weight plus somatic dry weight [95].

To provide an intuitive metric with respect to the magnitude of life history divergence, we conducted two discriminant function analyses (DFAs): one to evaluate separation of habitat types prior to the 2007 flood and a second one to evaluate separation of habitat types after the 2007 flood. We used a jackknife ('leave-one-out') sampling scheme as a cross-validation technique (*i.e*., each case is classified by the functions derived from all cases other than that case; see [54,56]. For both DFAs, *a priori*-probabilities were calculated based on group sizes. Furthermore, we tested for life history variation within each habitat type due to flooding by conducting cross-validation DFA as follows: all post-flood data were withheld, and a DFA model was built on pre-flood data only (training data set). The data from the withheld post-flood samples (testing set) were then inserted into the discriminant functions and assigned to the most parsimonious training data set category [96]. To accommodate for the potential effects of standard length (SL) and embryo stage on the other life history traits, we used residuals of a preparatory General Linear Model (GLM) as dependent variables. In this multivariate GLM, SL and embryo stage were included as covariates. The dependent variables for the GLM, and their residuals for the DFA, were female lean weight [g], female fat content [%], fecundity [number of offspring], reproductive allocation [%], embryo lean weight [mg], and embryo fat content [%]. For this preparatory multivariate GLM, all dependent variables and covariates were *z*-transformed to adjust for the influence of differential scale on measurements. All life history analyses were conducted using SPSS 16 for Mac (SPSS Inc. 2008).

## Results

### Genetic diversity

**Figure 4:**
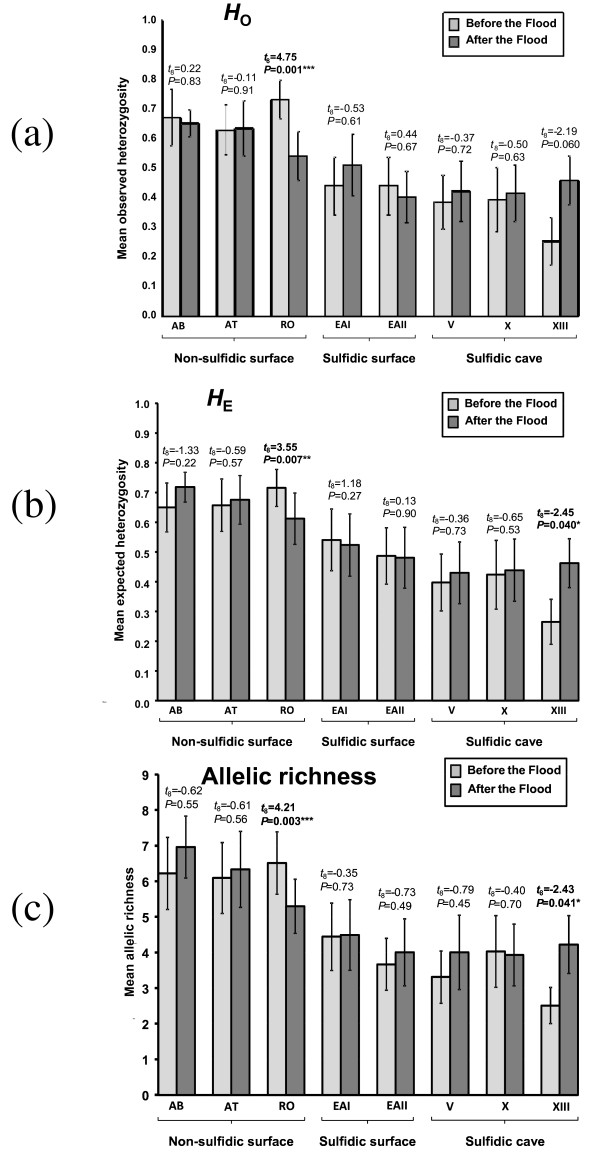
**Genetic diversity in eight populations of *P. mexicana *before (light gray) and after (dark gray) the flood (for populations code see legend to Fig. 1)**. Depicted are mean (± S.E.) values across loci for observed (*H*_o_, above) and expected heterozygosity (*H*_E_, middle), and allelic richness (*A*, below). Paired *t*-tests on locus-wise values within each population; significant comparisons are bold.

We found reduced genetic variability in the sulfur creek (El Azufre) population compared to non-sulfidic surface creeks, and a further decrease in the sulfidic cave (Cueva del Azufre). This pattern was observed before as well as after the flood (Fig. 4). Before the flood, genetic variability also varied within the Cueva del Azufre, with roughly equal values for observed heterozygosity (*H*_O_), expected heterozygosity (*H*_E_), and allelic richness (*A*) in chambers V and X, but distinctly lower genetic variability in the small rearmost chamber XIII. After the flood, however, values for *H*_O_, *H*_E_, and *A *in chamber XIII were similar to those of the other cave chambers (Fig. 4a-c). Indeed, pair-wise comparisons (paired *t*-tests) of pre- and post-flood data in chamber XIII indicated a statistically significant increase in *H*_E _and *A *after the flood (Fig. 4b-c), and a tendency toward significance (*P *= 0.06) in the case of *H*_O _(Fig. 4a).

Before the flood, individuals from chamber XIII were fixed at three of the nine microsatellite loci examined, but in all cases shared alleles from the other cave chambers after the flood. For example, at locus GAI29B, all fish from chamber XIII invariably were homozygous for an allele of 217 bp length before the flood, but after the flood also exhibited two additional alleles (225 and 227 bp), both of which had previously been detected only in chamber X. Likewise, all chamber XIII fish before the flood were homozygous for one allele (175 bp) at locus GTII33, while after the flood an additional allele (173 bp) was detected that had previously been found only in cave chamber V. Across loci, we found 12 different novel alleles to occur in chamber XIII after the flood that had previously been recorded exclusively in one or both of the other cave chambers, which was also reflected by large and significant pair-wise *F*_st_-values for the comparison of pre- and post-flood data (*e.g*., 0.173 in chamber XIII; Table 4). Altogether, this suggests extensive flood-induced population mixing among the different cave chambers within the Cueva del Azufre.

**Table 4:** Pair-wise genetic divergence (*F*_st_-values).

*F*_st_	AB	AT	RO	EAI	EAII	V	X	XIII
AB	0.019	0.022	0.018	**0.188**	**0.186**	**0.252**	**0.244**	**0.198**
AT	0.005	0.022	**0.031**	**0.219**	**0.222**	**0.283**	**0.270**	**0.235**
RO	0.002	0.006	**0.022**	**0.211**	**0.210**	**0.278**	**0.274**	**0.252**
EAI	**0.191**	**0.158**	**0.153**	0.000	**0.028**	**0.048**	**0.055**	**0.051**
EAII	**0.216**	**0.186**	**0.171**	0.027	0.015	**0.114**	**0.108**	**0.111**
V	**0.271**	**0.251**	**0.241**	**0.081**	**0.126**	**0.107**	0.015	-0.007
X	**0.270**	**0.235**	**0.234**	**0.059**	**0.149**	**0.099**	0.008	0.014
XIII	**0.349**	**0.326**	**0.315**	**0.155**	**0.189**	**0.066**	**0.134**	**0.173**

Below diagonal: before the flood, above diagonal: three months after the flood in fall 2007. Middle diagonal: *F*_st_-values for the comparisons of pre- and post-flood data-sets within each population. Statistically significant values after Bonferroni correction of *α*-levels are in bold typeface.

Genetic diversity was also affected by the flood in the central river of our study system (Río Oxolotán), where water currents were the strongest during the flood. Here, an impoverished genetic diversity was detected after the flood, and pair-wise comparisons (paired *t*-tests) for *H*_O_, *H*_E_, and *A *were highly significant in all cases (Fig. 4a-c).

### Genetic differentiation

The AMOVA on pre-flood data assigned 21.48% of the total variation to variability among populations (*F*_ST _= 0.215, *P *< 0.0001). This can be divided further into (high) variability among habitats (18.64%) and (low) variation among populations within these habitats (2.83%). After the flood, a slightly lower overall *F*_ST_-value was detected (*F*_ST _= 0.196, *P *< 0.0001). The proportion of the total variation among habitats was 17.86% and that among populations within habitats made up 1.74% of the total variation.

The Mantel test on pair-wise *F*_ST_-values explained 86.6% of the variation in post-flood *F*_ST_-values. Pre- and post-flood *F*_ST_-values were highly correlated (*r *= 0.931, *P *< 0.001), suggesting that general patterns of genetic differentiation were largely unaffected by the flood. Notably, there were significant effects of perturbation within the cave system, as significant genetic differentiation among the three sampled cave chambers (V, × and XIII) was detected before the flood (pair-wise *F*_ST_-values between 0.066 and 0.134), but not after the flood (*F*_ST_-values close to zero; Table 4). Moreover, significant *F*_ST_-values were detected for the comparisons of pre- and post-flood samples from the same locality in the case of chambers V and XIII (Table 4). This further substantiates the idea that the flood brought about increased population mixing (immigration and emigration) between different cave chambers.

The partial Mantel tests indicated that the effects of habitat type and distance, overall, did not differ prior and after the flood (Table 5). In both cases, pair-wise *F*_ST_-values were significantly lower between sites of the same habitat type than between sites of a different habitat type (pointing towards a pattern of 'isolation-by-adaptation'), while distance between sites did not have a significant influence on genetic differentiation.

**Table 5:** Partial Mantel tests on *F*_st_-values and number of migrants prior and after the flood.

	*r*	*P*
Pre-flood, pairwise *F*_st _(52.0%)		
**Habitat**	**-0.703**	< 0.001***
Distance	0.161	0.413
		
Post-flood, pairwise *F*_st _(68.7%)		
**Habitat**	**-0.751**	< 0.001***
Distance	0.352	0.064
		
Pre-flood, migrants (40.8%)		
Δ H_2_S	0.000	0.780
Δ Light	0.062	0.653
**Habitat**	**0.633**	< 0.001***
Distance	-0.063	0.661
		
Post-flood, migrants (63.4%)		
Δ H_2_S	0.000	0.747
Δ Light	0.068	0.615
**Habitat**	**0.790**	< 0.001***
Distance	-0.069	0.625

Percent variation (in parentheses), partial correlation coefficients, and *P*-values are provided for each Mantel test.

**Figure 5:**
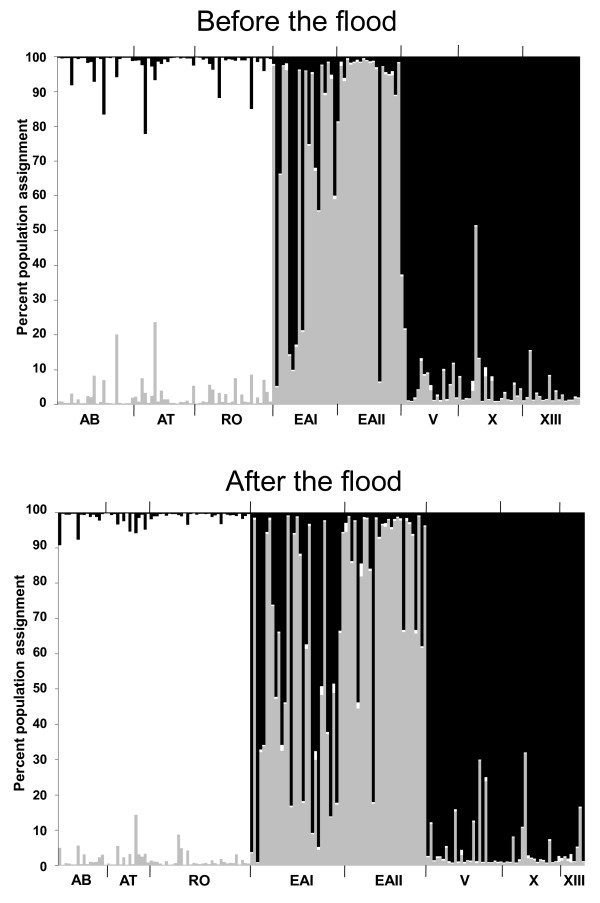
**Population assignment using STRUCTURE 2.3.2 Beta **[93]. For both data-sets [before (top) and after the flood (bottom)], *k *= 3 was recovered as the most likely number of genetic clusters.

Results from STRUCTURE for both data sets found the best statistical support for clustering all individuals into *k *= 3 groups according to habitat type (Fig. 5). While the first cluster (white) was composed of fish from the Arroyo Bonita, Arroyo Tres, and Río Oxolotán, the second cluster (gray) was made up mostly of El Azufre I and El Azufre II, and the third (black) of individuals from the cave. The occurrence of several cluster 3 (black) specimens also in El Azufre I (and to a lesser degree in El Azufre II) is indicative of some degree of unidirectional migration out of the cave into the adjacent sulfur creek (*i.e*., El Azufre I). This pattern was detected already before the flood, but appeared more prominent afterwards (Fig. 5). If we consider the highest probability of assignment (to the gray vs. the black cluster; see [97] for method) of any specimen in the sulfur creek, the percentage of specimens assigned to the black cluster had a tendency to increase from 15% (6/40) before the flood to 32% (18/57) after the flood (two-tailed Fisher's exact test: *P *= 0.093).

### Genetically detected dispersers

**Figure 6:**
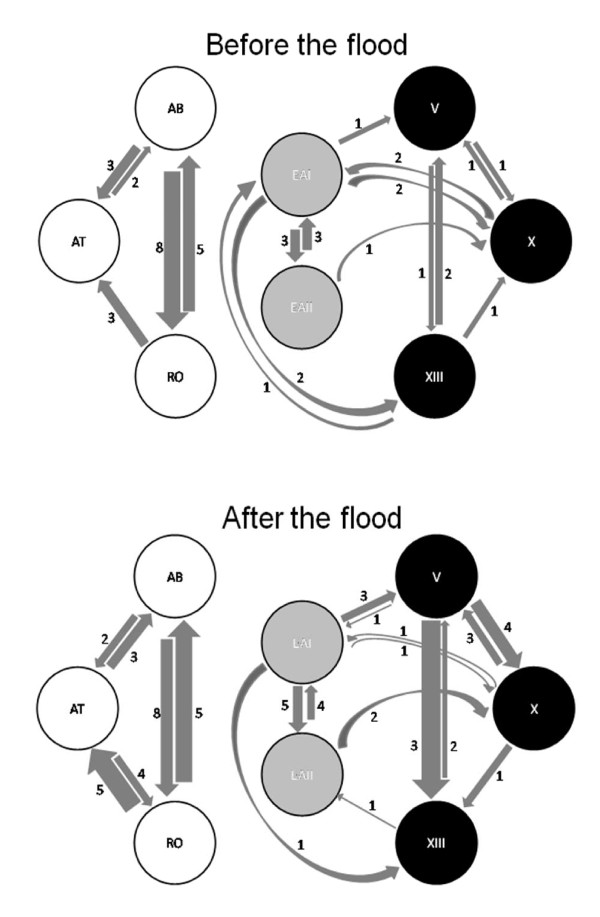
**Numbers of first generation migrants as calculated using GENECLASS 2.0 **[90]** before (top) and three months after the millennium flood in fall 2007 (bottom)**. The width of the arrows is proportional to the fraction of migrants in the 'recipient' population. Due to unequal sample sizes (see Tables 2, 3), absolute numbers of migrants may not always correspond with differences in proportions of migrants. 'Benign' surface habitats (white circles), sulfidic surface habitats (gray) and sulfidic cave (three cave chambers, black).

The STRUCTURE analysis already pointed towards a tendency for increased migration out of the cave into the sulfur creek in the course of the flood (see above and Fig. 5). According to our GENECLASS analysis, relative numbers of first-generation migrants were highest within the same habitat type and distinctly lower among different habitat types (Fig. 6). No migrants were detected between the clear water surface sites and the sulfidic surface or cave sites. Overall, migration patterns pre- and post-flood were strikingly similar in the GENECLASS analysis; with the exception of migration among the three cave chambers, which was increased after the flood (Fig. 6).

The Mantel test explained 71.1% of variance in numbers of migrants between sites, and pre- and post-flood numbers of migrants were highly correlated (*r *= 0.843, *P *< 0.001). The partial Mantel tests indicated that migration patterns were affected similarly by the predictor variables before and after the flood (Table 5). The only significant predictor of the number of migrants between sites was whether or not they belong to the same habitat type. There was no significant effect of distance, and migration was not more common from sulfidic to non-sulfidic (Δ H_2_S in Table 5) or from cave to surface habitats (Δ light).

### Population assignment using life history trait characterization

After the flood, females of two populations [sulfur creek (EA I and EA II) and non-sulfidic surface habitats (RA and AB)] suffered a strong decrease in female condition (*i.e*., female fat content), while embryo condition (*i.e*., embryo fat content) was decreased in all ecotypes. However, regardless of the flood event, the Cueva del Azufre and El Azufre were clearly distinct from the benign surface habitats (RA and AB) in that they produced few but large offspring before and after the flood (Table 6). Furthermore, there was a pronounced difference in offspring size and fecundity in the benign surface habitats. After the flood, AB females produced more but also larger offspring than did RA females before the flood (Table 6).

**Table 6:** Female life history traits for *Poecilia mexicana *from three different habitat types, sampled before and after the 2007 flood (Means ± S.E.).

Population	Sampling	SL [mm]	Female lean weight [g]^a^	Female fat content [%]^a^	Fecundity^a^	Embryo dry weight [mg]^b^	Embryo fat content [%]^b^	Reproductive allocation [%]^b,c^
CA (toxic cave)	pre-flood (*N *= 16)	35.94 ± 1.26	0.19 ± 0.02	6.07 ± 0.91	3.83 ± 1.69	8.17 ± 0.36	16.97 ± 1.01	12.51 ± 1.17
	post-flood (*N *= 21)	37.76 ± 0.91	0.19 ± 0.01	6.29 ± 0.80	3.88 ± 1.48	8.17 ± 0.31	14.55 ± 0.87	14.69 ± 1.02
EAI (toxic surface)	pre-flood (*N *= 8)	30.00 ± 0.71	0.29 ± 0.02	5.34 ± 1.36	11.59 ± 2.51	5.29 ± 0.53	22.12 ± 1.50	10.32 ± 1.74
	post-flood (*N *= 19)	32.05 ± 1.15	0.28 ± 0.02	1.79 ± 0.89	12.41 ± 1.64	4.46 ± 0.35	16.32 ± 0.97	13.84 ± 1.13
RA (benign surface)	pre-flood (*N *= 25)	33.84 ± 0.71	0.23 ± 0.01	13.54 ± 0.75	13.23 ± 1.38	2.61 ± 0.29	23.33 ± 0.82	10.89 ± 0.95
AB (benign surface)	post-flood (*N *= 37)	42.84 ± 1.33	0.28 ± 0.01	7.43 ± 0.69	21.84 ± 1.27	3.75 ± 0.27	19.30 ± 0.75	20.29 ± 0.87

^a ^Estimated marginal means from a GLM with SL as a covariate.

^b ^Estimated marginal means from a GLM with SL and Embryo Stage as covariates.

^c ^Reproductive allocation: Proportion of total dry weight which consists of developing embryos.

**Figure 7:**
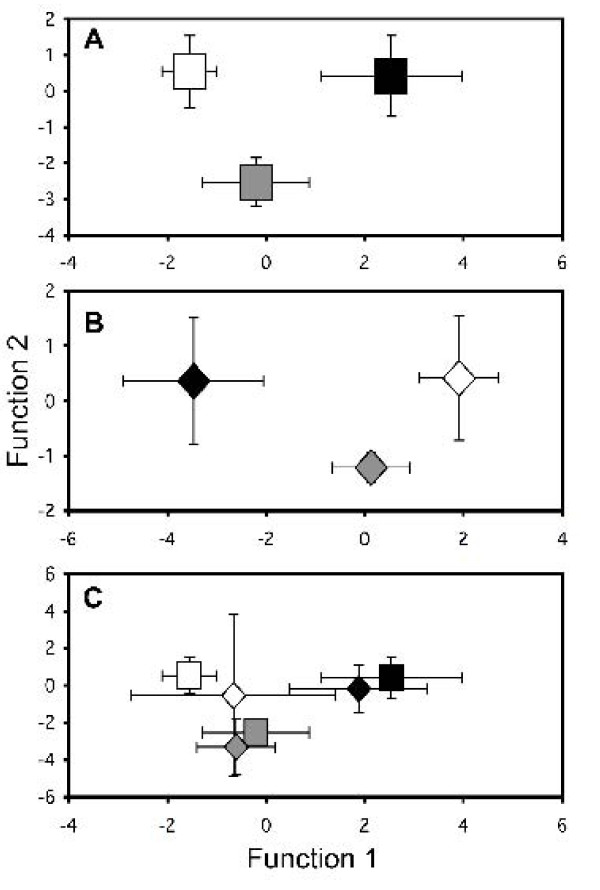
**Group centroids (± S.D.) of discriminant function analyses (DFAs) for separation of populations from different habitats based on female life history traits while controlling for female size (SL) and embryo stage**. (*A*) before and (*B*) after the flood in fall 2007. (*C*) cross validation analysis, with post-flood samples being used to validate the pre-flood classification. ('Black square') CdA pre-flood, ('Black diamond') CdA post-flood, ('Black square') EA pre-flood, ('Black diamond') EA post-flood, ('White square') RA pre-flood, and ('White diamond') AB post-flood.

Classification success for a separation by habitat type did not vary greatly before the flood (89.8%) and after the flood (87.0%), while the cross-validation DFA success was 66.2% (Fig. 7). Cross-validation success was highest in the El Azufre (100.0%), still good for the Cueva del Azufre (81.0%), but low in the non-toxic surface habitats (40.5%). In all cases, the most important life history traits for successful separation were fecundity and embryo lean weight (Table 6).

## Discussion

Extreme natural events can have profound effects on biological systems ranging from individuals to ecosystems [98,99] and--at least temporarily--reshape an organism's environment. However, the documentation of such events is often difficult, and precise data are rarely available to compare pre-disaster with post-disaster situations (but see [68,100]). Classic examples of well-studied catastrophic natural disasters include the explosion of Mt. Saint Helens in 1980, which stimulated a large body of research [101-105], or the impacts of seasonal hurricanes on species like the Puerto Rican Parrot [106] and *Anolis *lizards [107,108]. The impact of large scale floods on local adaptation and (parapatric) ecological speciation processes, however, has thus far not been examined.

Two months after the catastrophic flood in Tabasco in fall 2007, we found only little evidence for genetically detectible dislocation of individuals among the three divergent habitat types. In fact, habitat type, but not geographic distance, was the major predictor of genetic differentiation both before and after the flood. Furthermore, the pronounced genetic and life history differentiation among different habitat types (*i.e*., 'isolation-by-adaptation' [5,88]) was very similar for the pre- and post-flood datasets.

### Homogenization of locally adapted populations?

Our major question was whether the flood of 2007 has led to a homogenization of locally adapted populations, for which we found little evidence. A potential exception is the immigration of cave fish into the sulfur creek. It remains to be seen whether these immigrants are able to significantly contribute to the gene pool of the sulfur creek population or whether "cave alleles" will eventually vanish from the surface population with time (see below: *Mechanisms of selection against immigrants*).

Within the Cueva del Azufre, we did find that homogenization had occurred, since fine-scale (cave chamber-specific) genetic structure was lost after the flood in January 2008. While the small rearmost chamber XIII is usually separated by a 1.5 m waterfall from adjacent cave chambers, the flood in fall 2007 probably flooded the waterfall. Our current study provides several lines of evidence for the immigration of individuals into chamber XIII (*e.g*., several "newly" occurring alleles stemming from adjacent chambers). Hence, chamber XIII apparently is less separated from the other cave chambers than previously thought (*e.g*., [25,77]). The evidence for increased individual dislocation between different cave chambers (setting the stage for potential gene flow) also raises interesting new questions with regard to phenotypic divergence within the cave. From front to rear chambers, there is not only a morphological gradient with variation in eye and head size [77,109], but also heritable differentiation in opsin gene expression [110]. To date, it is not clear how the increased connectivity within the cave affected trait expression.

On the (slightly coarser) scale of the life history analyses, we observed several shifts from pre- to post-flood samples within habitat types (as exemplified by the lower classification success of the cross-validation DFA compared to the pre-flood-only DFA). At first sight, this might be interpreted as an indication of flood-induced migration/displacement between the habitat patches. Still, there was no breakdown of trait divergence between habitat types, since the extreme ecotypes (Cueva del Azufre and El Azufre) clearly remained distinct with respect to female life history traits even after the flood (post-flood DFA). In particular, highly plastic traits like female and offspring body condition (fat content) were decreased after the flood in almost all populations. This could either be a signal of the disturbance of the ecosystems due to the rising water levels and increased flow forces or regular seasonal variation of body condition [111]. Most importantly, the two traits known to be heritable in the Cueva del Azufre population (offspring size and fecundity [53,55]) did not show a flood signal at all, suggesting a lack of immigrants from the other habitat types.

In the surface habitats, however, the cross-validation classification success was by far the weakest. Potentially, this could be due to a plastic response to the flood, but it more likely represents general life history differences between the two surface habitats: RA is a large river, and fish were sampled in stagnant pools on a sandbank in the river, while AB is a small, fast flowing creek. Similar differences have been documented between guppy (*P. reticulata*) populations from river versus creek habitats [69], and we also have evidence for this scenario from recent studies on general life history differences between these two habitats derived from fish sampled in January 2009 (R. Riesch, unpublished data).

### Genetic diversity

We found a drastic impoverishment of genetic diversity in the Río Oxolotán after the flood. How can this pattern be explained? In the Río Oxolotán, water levels rose up to eight meters. Massive currents during the flooding probably washed away a considerable proportion of the total population. At first sight one might be tempted to interpret the observed reduction in measures of genetic diversity, above all observed heterozygosity (*H*_o_), as an indication of a genetic bottleneck. It needs to be recalled, however, that our post-flood sampling was done only two months after the flooding, while any mollies born after the flood would have needed more time to grow to adulthood (*i.e*., > 6 months under laboratory rearing conditions; R. Riesch, unpublished data). Hence, our samples originated from adult fish, and are probably not offspring of the few(er) remaining individuals in such (overall, genetically impoverished) populations. Reduced values of *H*_o _are, therefore, not as straightforward to explain as it may first seem. A possible scenario is that a big proportion of the population from our collection site in the Río Oxolotán was indeed swept away during the flood and was replaced almost entirely by fish from further upstream (the mountainous regions of Chiapas), and we hypothesize that fish in those upstream regions are characterized by lower genetic diversity. We plan to test this idea in the future by analyzing genetic diversity in more distant populations from all along the Río Grijalva drainage system.

Lower population sizes in the extreme (sulfidic) habitats generally coincide with a reduction in overall genetic variability (*i.e*., allelic richness and heterozygosity; for population sizes refer to [26,112]). Such small populations are inevitably prone to loss of genetic diversity over time due to genetic drift. Indeed, reduced genetic variability appears to be a typical feature of cave fishes and has been attributed to small population sizes, founder effects, and/or repeated genetic bottlenecks [113-117].

### Mechanisms of selection against immigrants

Particularly interesting in the study of ecological speciation are the mechanisms leading to and maintaining genetic differentiation, *i.e*., the question of how exactly divergent natural and sexual selection translate into reproductive isolation [9,10,118-120]. So, what are the potential mechanisms maintaining this small-scale genetic structuring even after such a catastrophic flood? Using reciprocal translocation experiments, we found natural selection against migrants between non-sulfidic and sulfidic habitats (with very high mortalities within 24 hours for fish from non-sulfidic waters when transferred into El Azufre and vice versa), whereas migrants between sulfidic cave and surface habitats did not exhibit increased mortality within the same time period [27]. A heritable basis to higher physiological sulfide-resistance in sulfide-adapted fish was confirmed by common-garden-rearing ([121]; reanalyzed in [28]). Further adaptations to survive under sulfidic, hypoxic conditions include plastic behavior, like aquatic surface respiration [122,123] and enlarged heads (a heritable trait) allowing for a larger gill surface area [26]. On the other hand, oxidative stress (*e.g*., due to down-regulated expression of antioxidant enzymes under hypoxia [124-126]) may explain the high mortality during translocation from sulfidic, hypoxic to normoxic sites ([27] for discussion).

But what about genetic differentiation between surface and cave habitats? This question is of particular importance because even before the flood some migrants/dislocated individuals from inside the cave into the sulfidic creek (El Azufre) were detected, and dislocation into El Azufre tended to increase due to the flood (Fig. 5). Given that genetic differentiation between both populations obviously remains stable over time ([25,26]; this study), strong selection against immigrants at the light/dark interface must be postulated. Generally, negative phototactic behavior was hypothesized to play a role for cave colonization [127,128]. Surface and cave fish could, theoretically, just differ in phototactic behavior, thus effectively preventing cave fish from venturing outside and vice versa. However, such a mechanism apparently plays no role in the Cueva del Azufre system, because both (at least lab-reared) surface- and cave fish are positively phototactic [129]. It has been demonstrated though that mollies in all habitat types experience predation by a giant water bug of the genus *Belostoma *[79,80,130], and a recent prey choice experiment under semi-natural conditions found that water bugs are more likely to capture dispersers, *i.e*., cave fish at the surface and surface fish inside the cave [57]. This may be due to sensory systems being maladaptive in the "wrong" habitat type. Cave mollies--living normally in perpetual darkness--have reduced eyes along with more elaborated non-visual senses like a hypertrophied head canal system of the mechano-sensory lateral line [25,49,77,109,110,121]. This allows for earlier detection of predators in darkness compared with the surface ecotype, while smaller eyes hamper predator detection in light [57].

In traditional models of ecological speciation, reproductive isolation evolves incidentally as a by-product of divergent natural selection [1-3,131]. But whenever divergent selection occurs among populations, there may also be direct (sexual) selection for premating isolation (*i.e*., reinforcement, [2,132]). In fact, females of both sulfidic populations (El Azufre and chamber II of the Cueva del Azufre) discriminate against immigrant male phenotypes during mate choice [27]. Altogether, selection against immigrants may indeed be a powerful mechanism facilitating speciation among locally adapted populations even over very small spatial distances, and we hypothesize that natural selection against immigrants plays the main role in maintaining the small-scale genetic structure.

## Conclusions

Both the population genetic analyses and the life history trait analysis (both as proxies for population genetic and quantitative genetic divergence) found the same pattern: increased flood-induced migration/displacement was found within the same habitat type, but not among ecologically divergent habitats (with the exception of slightly increased dislocation of cave fish into the sulfur creek). However, we did find some interesting patterns of flood-induced within-population homogenization (*i.e*., loss of small-scale genetic structuring within the cave). Overall, this supports the hypothesis that natural selection between habitat types in the Cueva del Azufre system is so strong that even severe ecosystem disturbances do not lead to a breakdown of the documented population differentiation. Hence, speciation processes in this and similar systems with presence of abiotic stressors (*e.g*., the Baños del Azufre system [54,133] may be more robust than those described for other systems in which differentiation is also based on ecologically based divergent natural selection [6,73,74].

## Competing interests

The authors declare that they have no competing interests.

## Authors' contributions

BH and CS carried out the microsatellite analyses, and RR conducted the life history analyses. MP, BH, CS, RR, MT and RT conducted the statistical analyses and wrote the first draft of the manuscript. MP, RR, MT, FJGL and IS were equally involved in field work and sample collection.

MP, RR, MT, FJGL, IS and RT were equally involved in implementing the project, and developing later drafts of the manuscript. All authors read and approved the final manuscript.
